# Exposure to extremely low frequency electromagnetic fields alters the behaviour, physiology and stress protein levels of desert locusts

**DOI:** 10.1038/srep36413

**Published:** 2016-11-03

**Authors:** Joanna Wyszkowska, Sebastian Shepherd, Suleiman Sharkh, Christopher W. Jackson, Philip L. Newland

**Affiliations:** 1 Department of Biophysics, Faculty of Biology and Environmental Protection, Nicolaus Copernicus University, Toruń, Poland; 2 Centre for Biological Sciences, University of Southampton, Southampton; 3 Engineering Sciences, University of Southampton, Southampton, UK

## Abstract

Electromagnetic fields (EMFs) are present throughout the modern world and are derived from many man-made sources including overhead transmission lines. The risks of extremely-low frequency (ELF) electromagnetic fields are particularly poorly understood especially at high field strengths as they are rarely encountered at ground level. Flying insects, however, can approach close to high field strength transmission lines prompting the question as to how these high levels of exposure affect behaviour and physiology. Here we utilise the accessible nervous system of the locust to ask how exposure to high levels of ELF EMF impact at multiple levels. We show that exposure to ELF EMFs above 4 mT leads to reduced walking. Moreover, intracellular recordings from an identified motor neuron, the fast extensor tibiae motor neuron, show increased spike latency and a broadening of its spike in exposed animals. In addition, hind leg kick force, produced by stimulating the extensor tibiae muscle, was reduced following exposure, while stress-protein levels (Hsp70) increased. Together these results suggest that ELF EMF exposure has the capacity to cause dramatic effects from behaviour to physiology and protein expression, and this study lays the foundation to explore the ecological significance of these effects in other flying insects.

Electromagnetic fields (EMFs) are pervasive in the environment, especially at extremely low frequencies (ELF 30–300 Hz) where they are given off by electrical appliances and overhead power lines. There have been many studies on the effects of exposure to EMFs. In humans there has been considerable interest in the negative health effects caused by high exposure levels^1^, with the European Union suggesting an occupational exposure level of 1 mT (Directive 2013/35/EU) to reduce the potential for damage. Insects, in common with many birds^2^,^3^, have a ‘magnetic’ sense including butterflies^4^, ants^5^, flies^6^, bees^7^ and cockroaches^8^. These insects are able to detect very low levels of static magnetic fields and use them to drive orientated movements. There is also evidence for different mechanisms of magnetoreception by organisms responsive to these low level static magnetic fields in the environment, including direct detection through either ferromagnetic crystal (Fe_3_O_4_) deposits^9^,^10^ or through cryptochrome molecules^6^,^11^.

Most studies on the effects of EMF in insects have focused on very low exposure levels, such as those generated by the Earths static magnetic field of <50 μT^10^ or fluctuating fields at ground level below power lines of <100 μT^12^. Surprisingly few studies have asked whether higher field strengths have an effect on behaviour, yet for flying insects much higher levels of EMFs are experienced in close proximity to the high voltage power lines where exposure levels under 400 kV transmission lines can be 0.6 mT at 1m from the conductor but as high as almost 14 mT at 1 cm from the conductor^13^. The consequence of this is that there is real potential for these higher fields strengths of 0.1 to 10 mT to impact on their behaviour and physiology. Moreover, few studies have analysed the effects of ELF EMF on the responses of individual neurones and synapses. In the crayfish Ye *et al*.^14^ analysed the effects of very high levels of magnetic fields on the responses of an identified interneurone in crayfish, the lateral giant (LG) interneurone, which is responsible for coordinating an escape response. In response to electrical stimulation of tail afferents the spikes and synaptic potentials in LG increased in amplitude during exposure leading the authors to suggest that these changes could lead to an increase in the sensitivity of LG. They also suggest that these effects were caused by the action of the magnetic fields on Ca^2+^ channels as they are in mice where EMF exposure leads to an increase in Ca^2+^ channels^15^.

At EMF levels less than 0.1mT exposure in human cell lines^16^, dipteran eggs^17^ and Planaria^18^ led to increased hsp70 levels during a single exposure, while in chick embryos repeated exposure led to reduced Hsp70 and cryoprotection^19^ suggesting an effect of treatment duration on Hsp70 levels. More recent studies on ELF-EMF over 1mT have been restricted to *in vitro* cell systems but have shown marked effects, including an increase in HSP70 transcription that leads to protection of chronic hypoxia^20^, and Ca^2+^ channel expression in neuronal synapses^15^ leading to effects on neuronal activity. In insects such as the locust stress or heat shock leads to a marked increase in Hsp levels^21^,^22^ that play a key role in thermoprotection. Thermal stress-induced heat shock responses have been described in locusts leading to an upregulation of hsps^23^. Barclay and Robertson^21^ showed that these stress responses could stabilize neuromuscular signalling during thermal stress, and that this could underlie thermoprotection of leg extensor muscle force output, possibly due to heat shock protein alteration of pre- or postsynaptic K^+^ channels^24^. In addition, in the locust flight system hsps reduce the thermosensitivity of synaptic delay and excitatory postsynaptic potential amplitude^25^. Stressors, including EMF, may activate a wide spectrum of interacting neuronal, molecular and neurochemical systems that underpin behavioural and physiological responses. Currently, however, we know nothing about the interaction between EMFs and heat shock protein expression in intact adult insects.

As a starting point to understanding how relatively high levels of EMF affect insects we take advantage of a well-studied insect, the desert locust, in which we know the components of the neural circuits controlling limb movements in detail^26^, we have well established methods to record from identified neurones^27^ and monitor muscle output^28^, and in which stress responses have been clearly identified^22^. In the locust we have the opportunity to also study the effects of high levels of ELF EMF at multiple levels in an animal where cognitive, behavioural, physiological and molecular effects can be analysed in detail to begin to understand how exposure to high levels of ELF EMF could impact behaviour.

## Results

### Effect of ELF-EMF on walking behaviour

**Figure 1:**
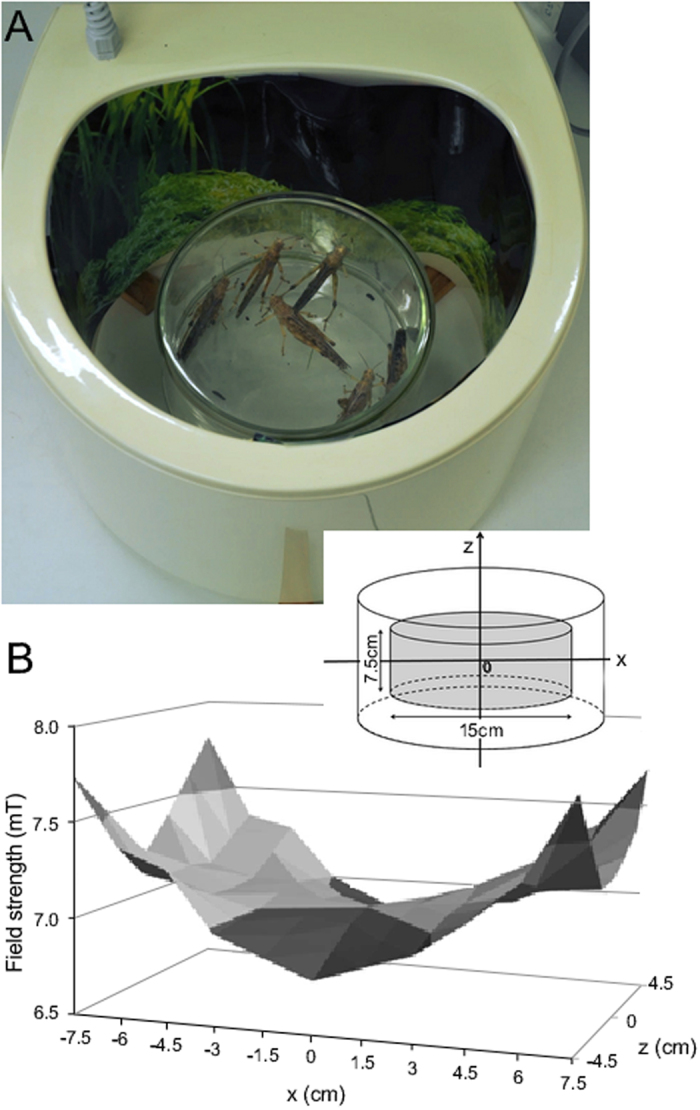
Exposure system. (**A**) Locusts in the magnetotherapy applicator/coil. (**B**) The average magnetic flux density distribution inside the solenoid along the Z and X axes. Inset shows the coordinate system.

**Figure 2:**
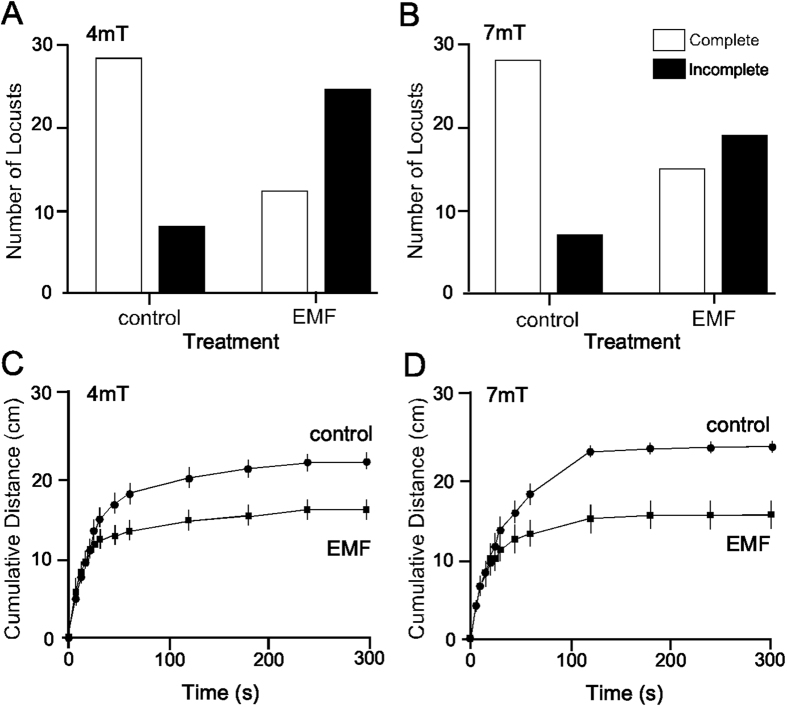
Effects of ELF EMF exposure on behaviour. (**A,B**) Graphs showing the number of complete and incomplete trials by locusts of the tunnel assay after 5 min at 4 mT and 7 mT respectively. At both exposure levels fewer locusts completed the tunnel assay following exposure to EMF compared to control animals. (**C,D)** Graphs showing the mean cumulative walking distance (±SEM) of locusts at 4 mT and 7 mT respectively. Locusts exposed to both 4 mT and 7 mT show reduced walking distances compared to controls.

To determine the effect of exposure to EMF (Fig. 1A) on walking behaviour the numbers of locusts completing the tunnel assay were compared between those exposed to EMF and their respective temperature matched controls. For both 4 mT and 7 mT EMF treatments the number of complete trials were less following exposure to EMF compared to controls (Fig. 2A). Following a 7 mT exposure 15 locusts completed the tunnel while 19 did not, whereas for matched temperature controls 28 locusts completed the tunnel while 7 did not (χ^2^ = 9.46, d.f. = 1, P = 0.0021). For a 4 mT exposure 12 locusts completed the tunnel while 24 did not, whereas for temperature matched control 28 completed the tunnel while 8 did not (χ^2^ = 14.40, d.f. = 1, P = 0.0001).

We then analysed the time taken for animals to complete the tunnel assay. With a 4 mT exposure there was no significant difference in the time taken for individuals to complete the tunnel (Welch corrected students’t test, t = 0.64, d.f. = 15.67, P = 0.53), with a mean completion time of approximately 90 s. Following a 7 mT exposure completion time (49.3 ± 8.6 s) was reduced compared to control animals (78.0 ± 10.7 s) such that EMF exposure significantly increased the speed at which locusts completed the tunnel (Welch corrected students’ t test, t = 2.08, d.f. = 40.54, P = 0.044).

Cumulative distance travelled in the tunnel assay also depended on EMF exposure. When EMF treatments were compared to their respective controls there was a decrease in the mean distance walked by locusts over the 5min time period for both a 4 mT exposure (Treatment factor, F_1,70_ = 4.603, P = 0.0354) (Fig. 2C) and a 7 mT exposure (Treatment factor, F_1,67_ = 6.478, P = 0.0132) (Fig. 2D). The effects of EMF on cumulative distance travelled was greater over time compared to controls, with exposed locusts covering less distance within the 5 min trial (4 mT exposure Interaction factor, F_12,840_ = 9.78, P < 0.0001 and 7 mT exposure Interaction factor, F_12,804_ = 10.88, P < 0.0001).

### Impact of EMFs on muscle force

**Figure 3:**
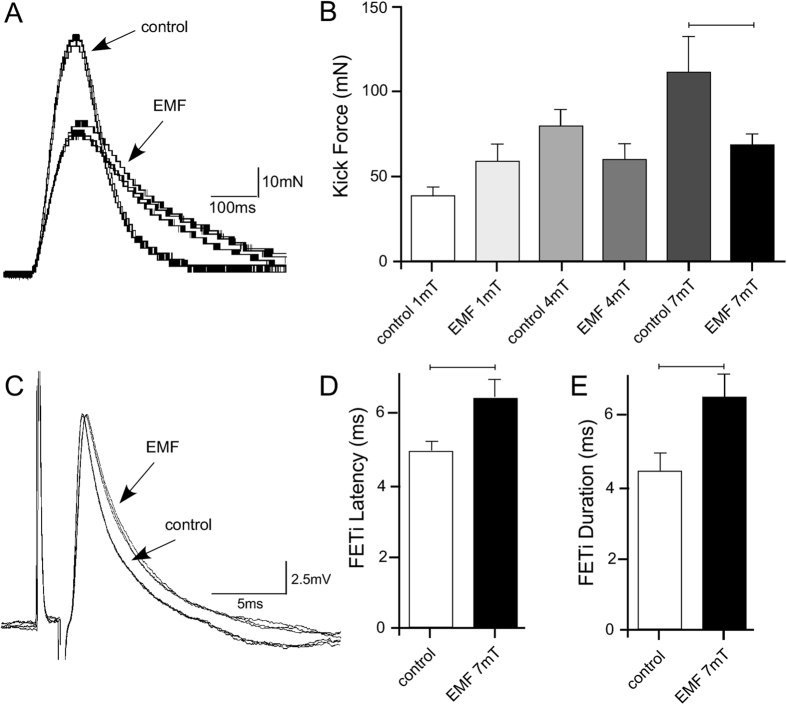
Effects of ELF EMF exposure on neuronal and muscle responses. (**A)** Superimposed sweeps showing kick force in control and treated animals exposed to ELF EMF at 7 mT. Kick force was reduced in treated animals. (**B)** Graph showing mean kick force (±SEM) in animals exposed to a range of field strengths. With a 7 mT exposure kick force was significantly reduced compared to temperature-matched controls (Students t tests). (**C)** Superimposed sweeps of intracellular recordings of FETi spikes evoked by antidromic stimulation of the ETi muscle. Spike latency and duration were both increased during exposure to 7 mT ELF EMF. (**D)** Graph showing a statistically significant increase in mean FETi spike latency (±SEM, Students t tests) following 7 mT exposure and (**E)**. graph showing a statistically significant increase in mean FETi spike duration (Students t tests) following 7 mT exposure to ELF EMF.

To determine the effect of ELF EMF exposure on muscle force, the hind leg ETi muscle was stimulated to produce a kick and the force of the resultant kick was measured^29^. Temperature matched control animals showed that with increasing control temperature (from 21 ± 1 °C for 1 mT exposure to 29.3 °C ± 1 °C for 7 mT exposure) there was an increase in kick force (Fig. 3A,B). With 1 mT and 4 mT EMF exposures there were no significant differences in kick force compared to respective temperature controls. For example at 1 mT the mean transformed kick force of exposed locusts was 58.7 ± 9.7 mN compared to 38.7 ± 5.4 mN for temperature matched controls (Students t test, t = 1.838, P = 0.08, d.f. = 22). At 4 mT the mean transformed kick force of EMF treated animals was 60.1 ± 9.0 mN compared to 79.5 ± 10.1 mN for temperature matched controls (students t test, t = 1.548, P = 0.129, d.f. = 46). Exposure to 7 mT for 24 hr, however, led to a significant decrease in kick force compared to the temperature-matched controls. The mean transformed kick force of chronically exposed animals was 68.4 ± 6.7 mN compared to 124.2 ± 19.9 mN for controls (students t test, t = 2.114, P = 0.040, d.f. = 48).

### Changes in neuronal responses

To test the effects of chronic EMF exposure on the nervous system, the dynamics of action potential and EPSP properties of the fast extensor tibiae motor neuron (FETi) were analysed (Fig. 3C–E). There was no significant effect of EMF exposure on action potential and compound EPSP amplitude in FETi (FETi spike: P = 0.33, t = 1.014, d.f. = 17; EPSP: P = 0.39, t = 0.88, d.f. = 17). The latency from muscle stimulation to the FETi action potential, however, increased from 4.98 ± 0.2 ms to 6.50 ± 0.5 ms after 7 mT EMF exposure (P = 0.03, t = 2.44, d.f. = 17) (Fig. 3D). By contrast there was no effect of EMF exposure on EPSP latency (P = 0.87, t = 0.16, d.f. = 17).

In addition, there was a significant increase in the duration of FETi action potentials in 7 mT EMF treated animals, increasing from a mean of 4.4 ± 0.5 ms to 6.5 ± 0.6 ms at the midpoint of the recovery phase (P = 0.03, t = 2.43, d.f. = 17) (Fig. 3E). There was no effect of EMF exposure on EPSP duration (P = 0.51, t = 0.67, d.f. = 17).

### The effect of ELF EMF on Hsp70 levels

**Figure 4:**
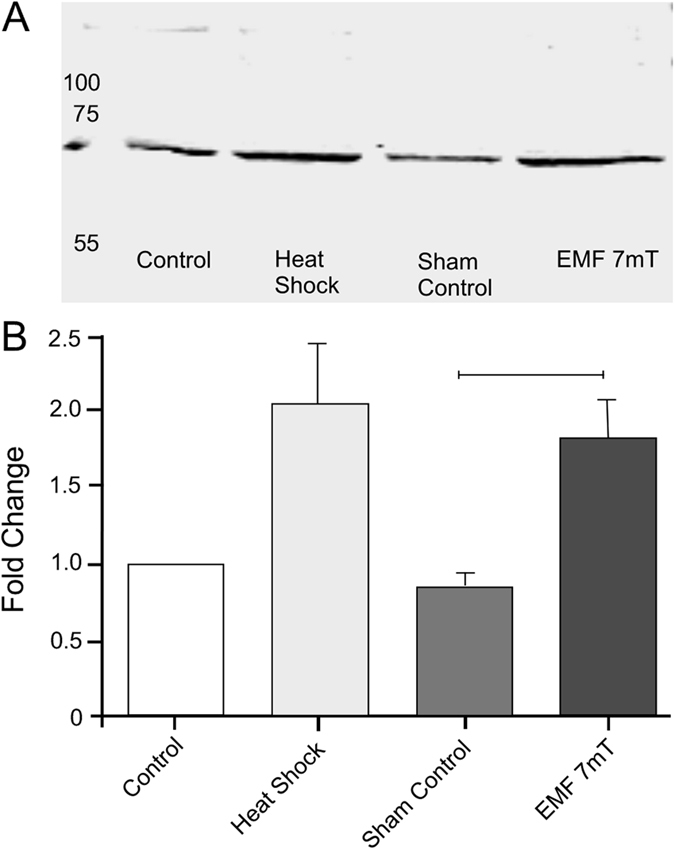
The effect of ELF EMF exposure on Hsp70 levels in the metathoracic ganglion. (**A**) An example of a Western Blot. (**B**) Graph showing mean fold changes (±SEM) in expression levels of Hsp70. All values are shown relative to the heat shock control treatment. There was a statistically significant increase in Hsp70 levels in animals exposed to 7 mT ELF EMF (Students t tests).

The effect of EMF exposure on molecular stress proteins was studied by measuring changes in the expression levels of the stress chaperone protein Hsp70 via Western-blotting (Fig. 4A,B). A positive heat-shock control led to an increase in protein levels by a factor of 2.1 ± 0.4 compared to the negative heat-shock control treatment. The two treatments tested (sham control and EMF 7 mT) were normalised to values relative to the negative control. Sham control Hsp70 levels were lower than control levels by a factor of 0.9 ± 0.1 whereas EMF 7 mT treatment levels resulted in Hsp70 levels higher than control by a factor of 1.8 ± 0.3. A students paired t test showed a significant increase in Hsp70 levels after a 7 mT EMF exposure (P = 0.026, t = 3.12, d.f. = 5).

## Discussion

Here we show that exposure to relatively high levels of ELF EMF, present around overhead power transmission lines, affect the behaviour, neuronal and muscular responses and levels of heat shock protein in the locust. Exposure to ELF EMF reduced walking in freely moving male and female locusts. Kick force was reduced during EMF exposure at 4 mT and 7 mT compared to matched controls, while the latency to the FETi spike was increased and spike duration lengthened during exposure. Finally, the levels of Hsp70 increased during exposure.

ELF EMFs significantly alter the normal walking behaviour of locusts, with distance travelled being reduced following exposure to EMF. Previous studies have suggested an acute effect of ELF EMFs on locust behaviour. For example, Bergh^30^ suggested that very-low frequency electromagnetic fields caused by storms increased the take-off rate of locusts that initiates flight while Clark^31^ found that locusts showed increased flight activity in proximity to storms. Lightning strikes from storms are known to generate ELF EMFs^1^ and while ELF EMF radiation from storms is very weak in comparison to the electrostatic forces generated^1^ the EMFs give rise to signals that can be detected over long distances^32^. Other insects have been found to be responsive to EMFs; for example Wijenberg *et al.*^33^ found ELF EMFs of 0.5–1 mT differentially affect various species of cockroaches, with both attractive and repulsive properties. It is clear therefore that relatively high levels of ELF EMF can alter insect behaviour quite dramatically and impact on their movements and therefore potentially their distribution.

ELF EMF exposure reduced muscle force output in the locust, increased the latency to spike in FETi and broadened the FETi spike; effects most pronounced at high levels of exposure. That EMF impacts on multiple levels in an organism is not surprising given that the nervous system functions via electrical signals and, as a result, is inherently susceptible to ELF EMFs^1^. ELF EMF exposure is known to induce electric fields, and currents, within organisms that can potentially excite neurons^34^,^35^ and it is possible that under a 24 hr exposure excitation of motor neurons could lead to a fatigued neuro-muscular system, resulting in poorer walking performance. Fatigue and adaptation can occur in response to repetitive stimulation in insect muscle in general^36^ and in the force output of the extensor muscle stimulated using similar methods described here^37^. Taniguchi and Tani^38^ also found EMF exposure increased the latency of motor-evoked action potentials in human erector spinae muscles while Bigland-Ritchie and Wood^39^ showed impaired action potential properties (including duration and delay, such as found in the locust) due to overstimulation of motor neurones, leading to a reduction in muscular force. These results provide striking similarities to those found in the locust and would explain why locusts do not complete the tunnel efficiently. With this in mind the link between disrupted FETi signalling and reduced ETi muscle force seems likely.

The effects of EMFs on the FETi action potential could be caused by any changes in Na^+^ and K^+^ levels, as these ions are critical for action potential generation. While few studies have explored the effects of ELF EMF on Na^+^ and K^+^ levels, however, Na^+^/K^+^ ATPase is known to be affected by ELF EMFs as the frequency of the electromagnetic fields used in this study (50Hz) is very close to the turnover rate of the enzyme^40^,^41^. There are many studies that show modified Na^+^/K^+^ ATPase activity when EMFs were applied^42^,^43^,^44^. Given that ELF EMFs are able to cause disruption to the process by which ion gradients are maintained then it is not surprising that FETi action potential are affected. Moreover, Barbier *et al.*^45^ showed that there was a significant change in Ca^2+^ influx following 50 Hz EMF exposure. Given the key role of Ca^2+^ in synaptic transmission and muscle contraction any changes caused by EMF would be likely to lead to changes in muscular forces as found in the locust. In other invertebrates, such as the crayfish, high levels of EMF effects the amplitude of action potentials in the lateral giant interneuron^14^ and intracellular Ca^2+^ levels. Given that high Ca^2+^ influx can be intrinsically linked to K^+^ efflux^46^ EMF related calcium changes could lead to disrupted Na^+^/K^+^ gradients, and an indirect ELF EMF effect on action potentials.

We found that ELF EMFs caused an increase in Hsp70 levels, which suggests that at the molecular level, stress processes are affected by short term exposure to high levels of ELF EMF, and these changes may underpin the other effects observed in this study. There is evidence that heat shock proteins can be upregulated from EMF treatments. For example, Li *et al.*^47^ found in *Drosophila* that short and long term 3 mT 50 Hz EMF treatments caused different expression of hsp22, hsp68, hsp70bc, hsp70, hsp60d. There are multiple pathways underlying induction of heat shock proteins, the first being a direct increase in expression of HSP70 through interaction with promoter region DNA^48^. Whilst this pathway is likely, with the other results found in this study, the potential for ELF EMF exposure to induce HSP70 expression through stress pathways is also likely. Heat shock proteins in insects (and other animals) are induced by a variety of molecular and/or physiological stresses^49^. As there are many pathways that could be affected to upregulate HSP70 expression from stress it is difficult to determine if a specific pathway may be affected. There is however evidence that muscle fatigue increases HSP70 expression in rats, after muscle tissue is electrically over stimulated^50^. This could potentially link with the results from other parts of this study as a molecular signal of the physiological effects that could lead to modified behaviour. There is also evidence from studies on rats^51^ that extremely high levels of static EMF can also lead to an increased HSP70 expression, however these levels of static EMF are many orders of magnitude greater than geomagnetic fields normally encountered in the environment and which many insects can detect^5^,^6^,^7^.

Here we have studied the effect of ELF EMF over 24 hr of exposure. The next step in our analysis is to determine the time course of effects and ask whether exposure to EMF could evoke an immediate response. While some responses are unlikely to be produced on immediate exposure, such as Hsp70 induction, whose time course can occur over minutes to hours that is clearly much longer than an immediate response^21^, other direct physiological effects on ion channels^45^,^46^ could well produce immediate behavioural responses and underpin avoidance movements to high levels of ELF EMF around overhead power lines which could then act as barriers to insect movement. It would also be pertinent to now consider how these high levels of EMF might impact on other insects that provide valuable ecosystems services such as key pollinators (honey bees) whose cognitive abilities are crucial in finding food. Any disruption to learning and memory could significantly impact on the valuable services they provide.

## Materials and Methods

Experiments were performed on adult desert locusts, *Schistocerca gregaria* (Forskål), aged from 4 to approximately 9 days post-moult and of both sexes, taken from a crowded colony at the University of Southampton. Locusts were fed on seedling wheat and oats and housed under a 12:12 light/dark cycle at 32 °C.

### EMF Coil and Field Characteristics

Electromagnetic fields (50 Hz) were produced using a 20 cm diameter coil (Elektronika i Elektromedycyna Sp. J.; Poland) composed of 282 turns of insulated copper wire (Fig. 1A). The coil and Variac power supply produced homogeneous, sine-wave alternating electromagnetic fields at 50 Hz and with intensities ranging from 0.1 to 8 mT. Field polarization was vertical so that field lines were perpendicular to the bottom plane of the chamber. The distribution of magnetic flux density within the coil varied along the length of the coil (Fig. 1B). Measurements of the fields for calibration were made using a Gauss meter (Model GM2, AlphaLab, Inc, USA).

### Exposure

Animals were exposed for 24 hr to control conditions or EMFs whilst kept in a glass chamber (15 cm diameter × 7.5 cm deep) located within the coil (Fig. 1A). 6 locusts were exposed together to ensure they remained in the gregarious phase^27^. Exposure to 1, 4 and 7 mT EMFs were analysed. 24 hr exposure to EMF led to heat within the coil that was dependent on the strength of the EMF. To control for this heat, temperature-matched controls were made using a hot plate under the coil to ensure the same temperature in the glass chamber for each respective EMF treatment. At 7 mT the temperature at the centre of the glass chamber increased to 29.3 °C ± 1 °C, while at 4 mT it was 24.5 ± 1 °C. At 1 mT there was no heating in the coil and control EMF treatments were compared at room temperature (21 °C). The temperature during experiments was monitored using thermocouples mounted under each exposure system to ensure that experimental conditions were similar, except for the presence of the ELF EMF.

### Walking behaviour

Walking behaviour was analysed in an open-top tunnel (30 cm × 10 cm × 8 cm) placed within a glass tank (30 cm × 40 cm × 60 cm) at room temperature (25 °C). Video recording was made from above. At one end of the tunnel a stimulus group of 5 gregarious locusts was placed in a mesh pot along with 7 g of seedling wheat and illuminated with a light source to act as an attractant. Individual test locusts were placed at the other end of the tunnel with their hind leg tarsi touching a line 5 cm into the tunnel. Walking was initiated by a single brushstroke with a fine tipped brush along tactile hairs on the dorsal tibia of one hind leg. Walking was recorded on a Sony HD video camera (HDR-CX115 Sony Handycam) and videos subsequently analysed offline. Locusts were given 5 min to walk the length of the tunnel, and for those that covered the entire length the trial was deemed to be complete, while for those that failed the trial was considered incomplete. The number of incomplete and complete trials were counted to calculate tunnel completion, while video analysis allowed measurements of cumulative distance travelled and completion time. In total 36 locusts were treated for each EMF level and control, and any reductions from 36 were due to losses over the 24 hr treatment period.

### Muscle force

To determine the effect of chronic ELF EMF exposure on muscle function the twitch force produced by the hind leg extensor tibia muscle (ETi) was analysed using previously established methods^28^,^29^. The ETi muscle was stimulated using 10 pulses at 0.1 Hz and resulting forces recorded. For 1 mT, 24 locusts were tested (6 female and 6 male for both control and EMF groups), for 4 mT 48 locusts were tested (12 female and 12 male for both control and EMF groups), and for 7 mT 50 locusts were tested (13 control locusts and 12 EMF treated locusts for each gender).

### Physiological Recordings

Intracellular recording of the hind leg fast extensor tibiae motor neuron (FETi) were made using methods previously described^27^,^52^. A pair of insulated 50 μm copper wires, exposed only at their tips, was implanted in the tibial extensor muscle of the hindleg and used to stimulate the ETi to produce antidromic spikes in FETi. The properties of the FETi spike and compound EPSP, resulting from synaptic input from campaniform sensilla^53^ were compared between temperature-matched control and EMF exposed groups using Spike7 software (Cambridge Electronic Design, UK). 8 control locusts, and 11 7 mT EMF-exposed locusts were tested.

### Stress Protein Levels

Metathoracic ganglia (containing the somata of FETi) were isolated from male locusts and the levels of Hsp70 detected via Western-Blotting. 7 mT EMF and respective sham control 24 hr treatments were compared, as well as positive and negative heat shock controls prepared using previously established methods^54^. Following treatment each locust was snap frozen in liquid nitrogen and the metathoracic ganglion was removed. Ganglia from three locusts were grouped in each sample to ensure adequate protein levels for blotting.

Samples were lysed in 2% sodium dodecyl sulfate (SDS) containing 1 × Halt™ protease and phosphatase inhibitor cocktail (Thermo-Fisher Scientific, UK). Sample concentrations were adjusted to 2.0 μg/μl with 5 × Loading Buffer (0.25% bromophenol blue, 0.5M DTT, 50% Glycerol, 10% SDS). Samples were then resolved by SDS-polyacrylamide gel electrophoresis (PAGE) on 7.5% acrylamide gels and transferred onto nitrocellulose membranes (Bio-Rad Inc.). Membranes were blocked in 5% non-fat dried skimmed milk (Marvel; Premier Foods, UK) in 1 × tris buffered saline (TBS) (Thermo-Fisher Scientific, UK) for 1 hr, and then washed in 1 × TBS 0.1%Tween-20 (Sigma-Aldrich, UK). Membranes were then incubated separately overnight in β-actin loading control (ab8224; Abcam^®^, UK) or Hsp70 (ab2787; Abcam^®^, UK) primary antibodies at a 1:1000 dilution in 5% BSA in 1 × TBS 0.1%Tween-20. Membranes were then washed and incubated in secondary antibody (IRDye^®^ 800CW 926–32210; Li-cor Biosciences, UK) at a 1:10000 dilution in 5% non-fat dried milk in 1 × TBS 0.1% Tween-20 for 1hr at room-temperature.

Membranes were imaged on an Odyssey scanner (Li-cor Biosciences, UK) using Image Studio version 4.0 (Li-cor Biosciences, UK). Hsp70 levels were normalised using the beta-actin loading control.

### Statistical analysis

Data were analyzed using Stat SPSS software (IBM Corp. Released 2013) and Graphpad Prism. Results are expressed as means ± standard error of the means (SEM). To determine whether exposure to EMF had an effect on tunnel completion the number of locusts that had complete trials and incomplete trials were totaled for EMF exposures and respective controls. A χ^2^ test was used to compare data in a 2 × 2 contingency table for both 4 mT and 7 mT exposures and high and low controls. To determine the effect of EMF exposure on completion time, a students’ unpaired t test using Welch’s correction for uneven variances was used to compare the mean completion time between EMF exposed locusts and control animals. Finally to determine the effect of EMF exposure on the cumulative distance travelled by locusts in the tunnel assay a two-way repeated measures ANOVA was used to compare the effects of ‘treatment’ (EMF exposure or respective control) and ‘time’ on the cumulative distance travelled along the tunnel.

To determine whether exposure to EMF had an effect on ETi muscle force output a students’ unpaired t test was used to compare mean muscle force output in locusts exposed to EMF with untreated control locusts. Similarly, to determine whether exposure to EMF resulted in a change in the FETi spike properties a students’ unpaired test was used to compare spike width and latency in EMF exposed animals with control animals. Finally, to determine the effects of EMF on Hsp70 levels a students’ paired t test was used to compare EMF exposed groups with control animals. In every case two-tailed Students t tests were used.

To determine the impact of EMFs exposure on muscle force data were square root transformed so that they were normally distributed and pairwise and unpaired students t-tests (Welch’s correction) were applied to the transformed data. All data were tested for normality and homogeneity, and the level of significance set at p < 0.05.

## Additional Information

**How to cite this article**: Wyszkowska, J. *et al.* Exposure to extremely low frequency electromagnetic fields alters the behaviour, physiology and stress protein levels of desert locusts. *Sci. Rep.* **6**, 36413; doi: 10.1038/srep36413 (2016).

**Publisher’s note**: Springer Nature remains neutral with regard to jurisdictional claims in published maps and institutional affiliations.
